# Clinical Advances of siRNA-Based Nanotherapeutics for Cancer Treatment

**DOI:** 10.3390/pharmaceutics13071009

**Published:** 2021-07-02

**Authors:** Dima Hattab, Amirah Mohd Gazzali, Athirah Bakhtiar

**Affiliations:** 1 Faculty of Pharmacy, University of Jordan, Queen Rania Street, Amman 11942, Jordan; deemahattab28@gmail.com; 2 School of Pharmaceutical Sciences, Universiti Sains Malaysia, Penang 11800, Malaysia; amirahmg@usm.my; 3 School of Pharmacy, Monash University Malaysia, Jalan Lagoon Selatan, Bandar Sunway 47500, Malaysia

**Keywords:** RNA interference, small interfering RNA (siRNA), nanoparticles, clinical trial

## Abstract

Cancer is associated with single or multiple gene defects. Recently, much research has focused on incorporating genetic materials as one of the means to treat various types of carcinomas. RNA interference (RNAi) conveys an alternative genetic approach for cancer patients, especially when conventional medications fail. RNAi involves the inhibition of expression of specific messenger RNA that signals for uncontrollable cell growth and proliferation, most notably with carcinoma cells. This molecular technology is promising as genetic materials allow us to overcome issues associated with chemotherapeutic agents including organ damage associated with severe drug toxicities. Nonetheless, vast challenges impede successful gene therapy application, including low tumor localization, low stability and rapid clearance from the blood circulation. Owing to the limited treatment opportunities for the management of cancer, the development of effective siRNA carrier systems involving nanotherapeutics has been extensively explored. Over the past years, several siRNA nanotherapeutics have undergone a period of clinical investigation, with some demonstrating promising antitumor activities and safety profiles. Extensive observation of siRNA-nanoparticles is necessary to ensure commercial success. Therefore, this review mainly focuses on the progress of siRNAs-loaded nanoparticles that have undergone clinical trials for cancer treatment. The status of the siRNA nanotherapeutics is discussed, allowing comprehensive understanding of their gene-mediated therapeutics.

## 1. Introduction

**Figure 1:**
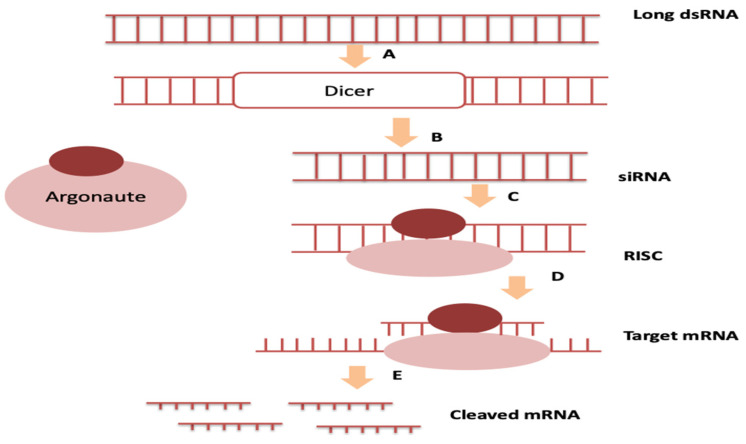
mRNA silencing by RNA interference. A–B. Cleavage of long dsRNA precursors by RNase III endonuclease Dicer to form smaller fragments, referred as siRNA. B–C. These short dsRNAs are subsequently unwound and the guide strand is assembled into RNA-induced silencing complex (RISC), that contains Argonaute protein (catalytic component) to cleave the passenger strand. C–D. RISC containing the guide strand will form complementary base sequence. D–E. The sequential will result in mRNA cleavage and degradation.

RNA interference (RNAi) is an innovative approach based on the delivery of noncoding double-stranded RNA (dsRNA) into cancer cells to trigger a homology-dependent degradation of the targeted messenger RNA (mRNA), leading to a selective and specific gene silencing [1,2]. The RNAi mechanism was first described by Fire and colleagues [3]. In contrast to most traditional pharmacological therapeutics that target proteins directly, RNAi-based drugs’ primary target is the mRNA. RNAi therapeutics of specific and selective nucleotide sequences apply non-coding RNA (ncRNA) from functional RNA molecules that leads to the targeted mRNA’s cleavage and subsequent downregulation of protein (Figure 1). Epigenetic-related ncRNA comprises small interfering RNA (siRNAs), microRNAs (miRNAs), piwi-interacting RNA (piRNAs), and long intervening noncoding RNA (lincRNAs) [4,5]. siRNAs are a large group of short noncoding RNAs, comprising 21–23 nucleotides. Synthetic siRNAs harness the naturally occurring RNAi mechanism in a constant and predictable manner, thus gaining appeal as therapeutics. Elbashir et al. revealed siRNAs abilities to instigate gene suppression in mammalian cells [6]. The siRNA pathway begins with the formation of siRNA from long double-stranded RNA (dsRNA) cleavage via Dicer complexes, followed by incorporation of siRNA into Argonaute 2 and RNAi-induced silencing complex (RISC). The siRNA is guided by RISC to induce complementary combination with endogenous mRNA according to base-pair binding. The downstream gene will subsequently be unable to express proteins and will be cleaved, therefore modulating cancer-related gene expression [7,8,9]. 

Nevertheless, naked siRNA poses many setbacks including short half-life and rapid clearance by the mononuclear phagocytic system (MPS) through opsonization and phagocytosis processes, as part of circulatory immune activity against foreign objects. The presence of biological barriers further impedes the delivery of siRNA into the targeted tissues, hindering its effectiveness in vivo. In order to expand the potential of siRNA, scientists have focused on developing and perfecting the gene delivery systems. In recent years, nanoparticles have been significantly embraced as a reliable gene carrier with good biocompatible and biodegradable properties [10,11]. Easy manipulation of nanoparticles characteristics enables the gene vehicle to improve the half-life of siRNA in the circulatory system [11,12,13]. The small dimension of conjugated nanoparticles further allows localization and distribution into its molecular targets within the cell, which in turn increases the tumor residence time through enhanced permeability and retention (EPR) effect [14,15,16]. 

Clinical application of siRNA-based nanotherapies in treating a wide variety of tumors poses numerous advantages; (1) siRNA nanotherapeutics can selectively and preferentially target any gene within the cancerous cells, especially the undruggable targets [17,18]; (2) they are readily fabricated and modified, in addition to having good safety and efficacy profiles with minimal off-target effects and immunogenicity [19,20]; (3) various siRNA therapeutics exhibited a promising antiproliferative and tumor growth suppression effect in vitro through the stat6 pathway and PLK1 [21,22], suppression of angiogenesis through inhibition of receptors including VEGFs and VEGFR-1 [23,24], or inhibition of tumor invasion and metastasis via chemokines CXCL8 and CXCL11 [25].

For more than twenty years, huge advancement in siRNA technology was accomplished; stable chemically modified siRNA moieties were constructed, and a wide variety of novel nanodelivery systems were acquired. The attention of researchers and the pharmaceutical industry is now focused on exploring siRNA-based therapies in clinical trials, which were initiated in 2012 and, to date, several synthetic siRNA-based therapeutics have been studied to treat recurrent and aggressive tumors, which are discussed in this review.

## 2. Investigational Phase and Study

To date, nine siRNA-based therapies have been investigated clinically (Table 1). Phase I trials have commenced for all siRNA anticancer nanotherapeutics: Phase I trials of CALAA-01 and DCR-MYC on patients with solid tumor, multiple myeloma and myeloma were terminated due to dose-limiting toxicities and sponsor decisions. ALN-VSP02, Atu027, TKM-PLK1, and siG12D LODER completed phase I favorably. A phase I trial of siRNA-EphA2-DOPC has begun recruiting samples but is still incomplete, whereas a trial on mesenchymal stem cell-derived exosomes with KRAS G12D is not yet recruiting. Four siRNA nanotherapeutics moved to phase II; Atu027 and TKM-PLK1 has completed phase II, while siG12D LODER is currently recruiting. A phase 2 trial on DCR-MYC was conducted on hepatocellular carcinoma patients but was subsequently terminated by the sponsor. No siRNA anticancer nanotherapeutics have undergone phase III trials as of May 2021. All the clinical trials at different phases are the first in human open-label trials. Participants’ harboring advanced stages and/or metastasis of diverse types of solid cancers that are not responding to standard of care (SOC) chemotherapy were recruited in these trials. 

### 2.1. CALAA-01

In 2008, CALAA-01 was the first siRNA-mediated nanoparticle that entered phase I clinical trials to treat solid tumors. CALAA-01 is an anti-RRM-2 siRNA incorporated within cyclodextrin-based nanoparticles coated with human transferrin. This vehicle selectively targets the transferrin receptors on the tumor cells’ surface, causing downregulation of ribonuclease reductase M2 subunit (RRM 2) [26]. RRM2 plays a significant role in DNA synthesis and replication. The M2 subunit is overexpressed, and subsequently, RR activity multiplies in cancer [27]. In phase Ia, CALAA-01 was administered intravenously with a dose range of 3–30 mg/m^2^ of siRNA for 15 patients with different cancer types. The early results were promising, with no dose-limiting toxicities (DLTs). Two patients receiving 30 mg/m^2^ experienced dose-limiting toxicities after two years that were untreatable even after reducing the dose to 24 mg/m^2^. In phase Ib, the CALAA-01 dose schedule was modified. Cancer patients were initially treated with 18 mg/m^2^, which patients tolerated well. In the subsequent cycle, with 27 mg/m^2^ dose, two out of five patients enrolled in phase Ib experienced DLTs [28]. Nevertheless, CALAA-01 was the first clinical trials involving siRNA therapeutics incorporated within a nanocarrier, and the first siRNA nanotherapeutics accumulated within the tumor cells in a dose-dependent manner. The trial was terminated in 2012 [26,28]. 

### 2.2. ALN-VSP02

In 2009, Alnylam Pharmaceuticals, in collaboration with Tekmira, fabricated novel dual siRNAs encapsulated within stable nucleic acid-lipid particles (SNALP) (ALN-VSP02). ALN-VSP02 contains two siRNAs silencing kinesin spindle protein (KSP) and VEGF expression. Both KSP and VEGF expressions are overregulated in patients with cancer. Downregulation of KSP and VEGF expressions results in inhibition of both tumor cell proliferation and angiogenesis [29]. A phase I trial included the dose-escalation phase and expansion phase. In the dose-escalation phase, 30 patients with hepatic and/or extrahepatic tumors were enrolled for intravenous ALN-VSP02 at a dose ranging from 0.1 to 1.5 mg/kg. The phase was completed with no safety concerns. Subsequently, after four cycles of ALN-VSP02, well-tolerated patients underwent the expansion phase at either 1 mg/kg or 1.25 mg/kg. In this trial, one patient with endometrial cancer and hepatic metastasis experienced a complete response after 50 doses of ALN-VSP02. Approximately 12–18 months of tumor stabilization occurred in three other patients with either renal cell cancer or pancreatic neuroendocrine tumor. Owing to the small sample size of the ALN-VSP02 study, heterogeneity of tumors, and different chemotherapeutics and anti-VEGF therapies used, no correlations were established between the molecular signaling pathways’ suppression and patients’ clinical response [30,31,32].

### 2.3. Atu027

**Figure 2:**
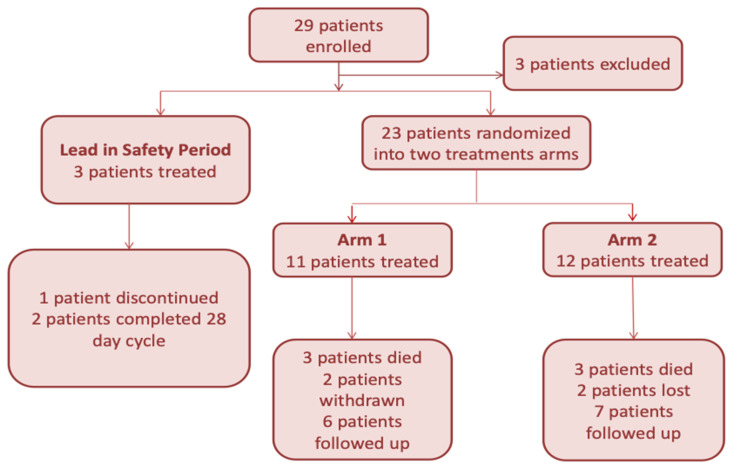
Phase Ib/IIa study design and assessment of patients receiving Atu027 and gemcitabine therapy.

**Figure 3:**
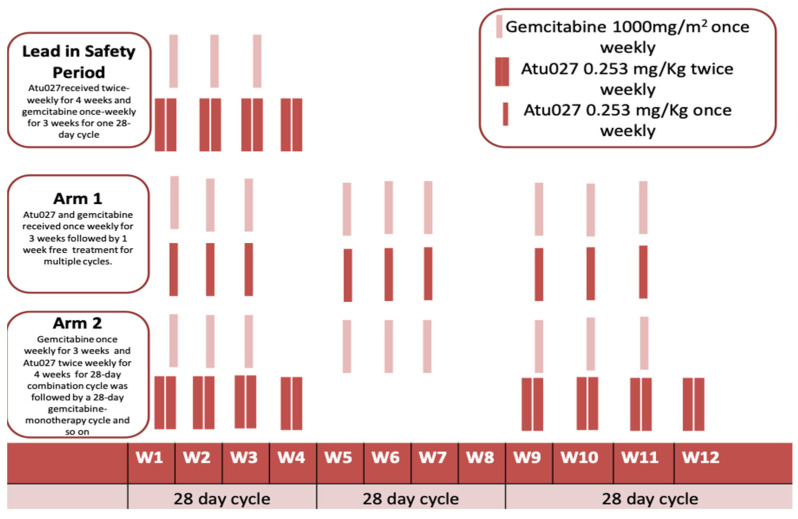
Dosage regimen in the safety cohort, arm 1 and arm 2 of phase Ib/IIa trial.

Atu027 is a siRNA-based lipid nanoparticle that has the ability to knockdown the expression of protein kinase N3 (PKN3). Elevated PKN3 expression is associated with enhanced angiogenesis and tumor metastasis; consequently, silencing PKN3 expression leads to the inhibition of angiogenesis and tumor invasion [33,34]. A phase I clinical trial investigation of on Atu027, via a cationic lipoplex nanocarrier known as AtuPLEX, was developed by Silence Therapeutics for patients with advanced solid tumors. Atu027 was administered intravenously on 24 patients at doses of up to 0.18 mg/kg. Two patients experienced either disease stabilization or pulmonary metastasis regression; the patients were further treated with Atu027 until the cancer became progressive. The phase I trial was completed with no DLTs [35,36]. Atu027 response was significantly greater in patients with pancreatic neuroendocrine tumor [37]; therefore, a phase Ib/IIa trial was launched in 2013 to investigate the safety-efficacy profile of Atu027 in combination with gemcitabine in advanced pancreatic adenocarcinoma patients (Figure 2). Twenty-nine patients were randomly enrolled into two different Atu027 schedules. In one group, patients were treated with Atu027 and gemcitabine once weekly for three weeks; patients in the second group were treated with gemcitabine once weekly and Atu027 twice weekly for three weeks followed by one drug-free week (Figure 3). The 28-day cycle was repeated until the appearance of significant toxicities or disease progression. Phase Ib/IIa revealed that both regimens were well tolerated and the twice-weekly dosing superseded the once-weekly dosing [38].

### 2.4. TKM-PLK1

Tekmira Pharmaceuticals developed siRNA incorporated on a SNALP nanocarrier to treat advanced solid tumors that are involved in primary or secondary liver cancer, called TKM-PLK1/TKM-080301. TKM-PLK1 targets the polo-like kinase 1 (PLK1). PLK1 attracted scientists’ attention as it plays a pivotal role in cell cycle proliferation in normal cells, and it is overexpressed in a wide variety of cancerous cells and, therefore, contributes to poor cancer prognosis [39]. TKM-PLK1 leaped into human trials by recruiting patients with lymphoma and other solid tumors including advanced adrenocortical carcinoma (ACC), and hepatocellular carcinoma (HCC). The dose-escalation phase studied the effectiveness and safety of TKM-PLK1 in patients with lymphoma or advanced solid tumors at doses ranging from 0.15 to 0.9 mg/kg weekly followed by one drug-free week. TKM-PLK1 showed an antitumor effect at the studied doses and a favorable toxicity profile at doses of less than 0.9 mg/kg [40,41]. In 2010, a phase 1 trial was initiated on 16 patients harboring advanced ACC. Quantities of 0.6 or 0.7 mg/kg of TKM-PLK1 were parenterally administered once weekly for three weeks, followed by one drug free week, for up to 18 months. It was found that TKM-PLK1 is safe at the interventional doses with moderate antitumor activity. The trial was completed in 2014 with indication that further studies are obligatory [42]. The safety–toxicity profile of hepatic artery infusion of TKM-PLK1 was investigated in 2011 in patients with metastatic liver from colorectal, breast, ovarian, gastric, and pancreatic cancers. The trial was completed in 2012, but the results were not reported [43,44]. Another clinical trial was performed in 2014 that investigated TKM-PLK1 safety, tolerability, pharmacokinetics parameters, and antitumor activity in patients with advanced HCC. In the phase I trial, twelve patients were recruited in three cohort studies, all receiving intravenous infusion of 0.3, 0.6 and 0.75 mg/kg TKM-PLK1. It was found that 0.75 mg/kg is the maximum tolerable dose with an acceptable toxicity profile. A subsequent expansion study that enrolled thirty-one patients with 0.75 mg/kg TKM-PLK1 reported unsatisfactory antitumor effect of TKM-PLK1 as monotherapy, despite being well-tolerated [45,46].

### 2.5. siG12D LODER

siG12D LODER is the first intra-tumoral administered siRNA anticancer nanotherapy which was clinically evaluated in 2011. siG12D LODER is a slow and prolonged-release local delivery system comprising LOcal Drug EluteR copolymer (LODER) and embedding anti-KRAS siRNA (siG12D) [47]. Local Drug EluteR (LODER) is a biodegradable polymer developed by Silenseed Ltd. as a nanocarrier for siRNA therapeutics. A comprehensive in vitro study on siG12D LODER, conducted by Ramot et al., revealed a favorable safety profile with no local and systemic toxicities after repeated administration [48]. siG12D LODER targets G12D-mutated KRAS in patients with pancreatic ductal adenocarcinoma (PDA). As mutant KRAS is associated with increased pancreatic cancer cell proliferation and tumor growth, knockdown mutant KRAS expression will result in suppression of PDA cell growth [47]. In the phase I trial, the safety and effectiveness of siG12D LODER were investigated on 15 patients with non-metastatic advanced PDA. Patients were separated into three dose cohorts, receiving a single dose of 0.025 mg, 0.75 mg, or 3 mg siG12D inserted directly into the pancreas by endoscopic ultrasound (EUS) biopsy injection concomitantly with SOC chemotherapy (weekly IV gemcitabine, or a combination of gemcitabine, erlotinib, and oxaliplatin—FOLFIRINOX). The three interventional cohorts’ safety data were favorable, with no dose-limiting toxicities reported even at 3 mg siG12D LODER. Superior clinical responses were also demonstrated in the combination of siG12D LODER and SOC chemotherapy [49]. SiG12D LODER passed phase I successfully, and a phase II trial was initiated in 2017. In phase II, 80 patients were assigned into two interventional groups. Patients with unresectable local PDA received either repeated doses of 3 mg siG12D LODER along with chemotherapy (gemcitabine and nab-paclitaxel) every four months or received chemotherapy treatment alone. This phase II trial is currently underway.

### 2.6. KRAS G12D

Novel KRAS-siRNA-based nanotherapeutics were recently developed by incorporating G12D mutated KRAS-siRNA within exosomes (iExosomes). CD47 functionalized exosomes are extracellular nanovesicles derived from human foreskin fibroblast-like mesenchymal stem cells (MSC). They are characterized by high circulatory retention time with good cellular internalization via oncogenic RAS enhanced macropinocytosis in cancerous pancreatic cells, in addition to specific targeting of cancerous cells with oncogenic KRAS G12D mutation [50,51]. CD47 functionalized exosomes loaded with anti-KRAS G12D are promising delivery platforms to silence KRAS G12D expression and reduce pancreatic tumor burden exclusively in G12D-mutated KRAS-harbored cancerous pancreatic cells. Preclinical studies demonstrated that KRAS siRNA-engineered iExosomes reduce KRAS expression in patient-derived PDA xenografted mice, resulting in compromised cancerous cell proliferation, enhanced apoptosis, inhibited metastasis, and increased overall survival with no cytotoxic effects [52,53]. Treatment via mesenchymal stem cell-derived exosomes with KRAS G12D siRNA is intended for patients with metastatic pancreatic cancer or PDA with KRAS G12D mutation, but Phase I trial for this treatment is not yet recruiting.

### 2.7. DCR-MYC

DCR-MYC (also known as DCR-M1711) is a Dicer substrate siRNA (DsiRNA), which was fabricated by Dicerna Pharmaceuticals. This DsiRNA is encapsulated within an EnCore^TM^ lipid nanoparticle. DCR-MYC targets c-Myc overexpressed cancerous cells, resulting in the silencing of c-Myc expression and inhibiting cell proliferation and growth in different types of cancers. DCR-MYC commenced phase I trial in 2014, in which cancer patients (solid tumors, multiple myeloma, Non-Hodgkin’s Lymphoma, and pancreatic neuroendocrine tumors) were enrolled for intravenous treatment of DCR-MYC once a week for two weeks, followed by a drug-free week. DCR-MYC showed a favorable safety profile and promising siRNA-based nanotherapeutics targeting c-Myc. In 2015, DCR-MYC was investigated in a phase Ib/II trial that recruited patients with advanced HCC who were not responding to SOC sorafenib therapy [54]. Despite being the first siRNA therapeutic regimen targeting c-Myc that was evaluated clinically, the preliminary clinical results did not meet the researchers’ expectations; hence, the sponsor decided to terminate the DCR-MYC trials.

### 2.8. EphA2-siRNA-DOPC

EphA2-siRNA-DOPC is one of the most recent anticancer siRNA-mediated nanotherapeutics to be investigated clinically. EphA2 is a type of tyrosine kinase protein receptor that is expressed abundantly in embryos and, to a lesser extent, in adults, mainly at the surface of epithelial cells. Several studies revealed that EphA2 is selectively upregulated in many cancers such as breast, pancreas, prostate, lung, and, most importantly, ovarian cancer. It influences cell growth, invasion, metastasis, and angiogenesis, and increases the tumor burden. EphA2-siRNA was incorporated into liposomal nanoparticles (DOPC) called EPHARNA (EphA2-siRNA-DOPC), specifically targeting EphA2 expression in epithelial cell-mediated tumors [55,56,57]. In vitro and in vivo studies demonstrated that EPHARNA has an anti-angiogenic effect and reduces tumor growth dramatically. Moreover, remarkable tumor growth inhibition was identified with simultaneous administration of EphA2-siRNA-DOPC and paclitaxel [56]. In vivo toxicological studies revealed that DOPC nanoliposomes exhibited no observed adverse events at a dose range of 75–225 mcg/kg following single administration, and two administrations resulted in insignificant toxicities [57]. In 2015, EphA2-siRNA-DOPC entered a phase I trial that recruited patients with advanced metastatic solid cancer who were receiving intravenous EPHARNA twice weekly over 2 h. The trial is still ongoing [58].

### 2.9. NBF-006

NBF-006 is a lyophilized LNP encapsulating siRNA targeting glutathione-S-transferase P (GSTP). GSTP is a well-known regulator of RAS signaling pathway proteins and it is extensively over-expressed in various types of cancer, especially KRAS mutant cancers including lung, colorectal and pancreatic cancer. A pre-clinical study performed on a KRAS mutant non-small-cell lung cancer (NSCLC) xenograft model receiving NBF-006 showed that NBF-006 was well tolerated by the animal models; 70–80% of the administered dose was internalized by the tumor tissues, causing significant tumor suppression and prolonged survival [59]. Consequently, NBF-006 entered a phase 1 trial that investigated the GSTP knockdown impact on patients with NSCLC, colorectal or pancreatic cancer with or without KRAS mutation. NBF-006 was intravenously administered once weekly for 4 weeks and repeated every 6 weeks. Patients with KRAS mutant cancers, however, underwent a longer treatment duration in the human trials [60].

## 3. siRNA-Mediated Nanotherapeutics in Clinical Trials

### 3.1. Administration and Distribution

All siRNA therapeutics were encapsulated within nanocarriers, no naked siRNA was parenterally-administered for the clinical study (Table 2). Miscellaneous artificial nanocarriers were used as platforms to deliver siRNA to their targeted destination, including lipid nanoparticles and polymer-based nanoparticles: ALN-VSP02, Atu027, TKM-PLK1, DCR-MYC and NBF-006 were embedded in lipid nanoparticles, and siRNA-EphA2-DOPC was encapsulated in liposomes, whereas CALAA-01 and siG12D LODER were loaded in polymer-based nanoparticles. Only mesenchymal stem cell-derived exosomes with KRAS G12D were incorporated in natural extracellular nanovesicles [61].

It is documented that only 0.7–0.9% of the administered nanoparticle dose was internalized by the cancerous cells, indicating low cellular uptake efficiency and high off-target cytotoxicity [62]. However, the superiority of exosomes in their siRNA delivery and specific targeting into PADC cells was reported by Kemarkar et al. [53]. This study revealed that, compared to liposomes, CD47 functionalized iExosomes are highly efficient at escaping phagocytosis and being retained in circulation for up to 3 h post-intraperitoneal injection. iExosomes were significantly accumulated in the G12D mutated KRAS mediated PDA cells, causing impairment of tumor cell proliferation, ameliorating apoptosis and tumbling the tumor even after treatment cessation. Although iExosomes uptake was not allied to CD47, cellular internalization of iExosomes was strikingly higher in mutant pancreatic cancerous cells [53]. iExosomes are widely distributed into the liver, colon, spleen, lung, and brain. In contrast, synthetic nanoparticles’ biodistribution is restricted to specific organs such as the liver, spleen, and kidneys for polymer-based nanoparticles, and essentially restricted to the liver for lipid nanoparticles [63]. Nanoparticles’ biodistribution is fundamental for both tumor targeting and dose-limiting toxicities. LNP delivery systems are highly efficient for treating liver diseases, but frequent administration may result in hepatotoxicity [64].

CALAA-01 and MSC-derived exosomes with KRAS G12D siRNA are the only siRNA nanotherapeutics that employ targeted nanoplatforms. CALAA-01 is functionalized with human transferrin ligand targeting highly-expressed transferrin receptors on tumor cells. Although it has no antitumor effect, the enclosure of transferrin at the surface of CALAA-01 enhances its cellular internalization activity. Post-dosing biopsies of the tumor sections on three patients receiving different dosages of CALAA-01 by Davis et al. revealed stained regions, indicating the presence of nanoparticles solely in the tumor cells. The number and the intensity of the stain were proportional to the CALAA-01 dose and the treatment duration. Efficient intracellular delivery of the targeted nanoparticles resulted in marked inhibition of RRM2 expression in post-dosing biopsies [28]. Nonetheless, many disadvantages were linked to the presence of transferrin, primarily including destabilization of the drug product and elicitation of major DLT in two out of five patients enrolled in the trial. CD47 ligand at the exosomes’ surface plays no role in the uptake of MSC-derived exosomes into tumor cells. CD47 transmembrane protein protects the nanovesicles from being phagocytized by binding to the signal regulatory protein alpha (SIRPα) and stimulating “do not eat me” signals, thus decreasing its systemic clearance in order to enhance the delivery to the tumor site. As CD47 ligands are natural components of the exosomes, short and long-term administration will not be accompanied by major adverse events, and they are impervious to most transferrin-mediated drawbacks including instability and high manufacturing costs of complexes.

LODER and NBF-006 are the only biodegradable delivery systems (LODER is polymer NP but NBF-006 is LNP) in the clinical trials. Biodegradable nanoparticles are safer than non-biodegradable ones; following administration, they are disassembled into smaller fragments that are promptly cleared from the circulation [65,66], as opposed to the non-biodegradable nanoparticles that have a higher potential to induce immune response and off-target toxicities. Essentially, preclinical safety studies are required to evaluate the secondary biodegradable fragments. Ramot et al. examined LODER’s safety in vivo and found that LODER forms a capsule of thin fibrotic tissue with a thin layer and macrophages after intracellular siRNA release multinucleated giant cells at the interface between the capsule and LODER. Capsule formation was not linked with any potential side effects and did not affect the pharmacokinetics parameters, hence affirming its safety for local and systemic administration [48].

ALN-VSP02 is the only investigational siRNA anticancer nanotherapeutic that incorporates two distinct siRNAs that target the expression of two genes (KSP and VEGF). Integration of two or more siRNAs targeting multiple genes at different molecular signaling pathways improves the therapeutic effect without increasing the toxicity. Yuan et al. revealed that simultaneous delivery and suppression of multiple gene/protein targets (KRAS, PIK3CA, and PIK3CB) inhibits tumor growth, significantly surpassing the inhibition caused by administration of high doses of a single anti-KRAS siRNA [67]. The inhibitory activity may explain the patient’s complete response achieved in the ALN-VSP02 trial. The best response associated with CALEA-01, Atu027, TKM-PLK1, DCR-MYC, and siG12D LODER administration was disease stabilization and partial response.

Chemical modification of siRNA poses great advantages including high stability in serum, good RNAi access and efficient immune escape. The modification of siRNA is often presented at the 5′ or 3′ terminus, sugar or nucleobase of the siRNA structure, whereby the fundamental requirement of efficient modification of siRNA the improvement of serum stability without affecting its silencing activity [68]. siRNAs in ALN-VSP02 and Atu027-related studies were chemically modified prior to dose administration. Most patients receiving ALN-VSP02 showed an immune-stimulating effect associated with short-term elevation of cytokine levels despite chemical modifications of the double-stranded siRNA in ALN-VSP02. No significant changes in cytokine levels were observed following Atu027 dosing.

Various concerns have to be deliberated once chemical modifications are employed; firstly, all investigational siRNA anticancer contents were protected within nanoparticles, preventing degradation by serum nucleases. Secondly, all RNAs that were used clinically to induce gene silencing are double-stranded siRNAs. It was demonstrated by Sioud that double-stranded siRNAs are less immunogenic than single-stranded ones [69]. Thirdly, some delivery systems are less likely to be immunogenic than others. Moyano et al. demonstrated that the immune-stimulating effect is highly-correlated with the hydrophobicity of the nanoplatforms [70]. Fourth, chemical modifications proposed to improve the intracellular stability of anticancer siRNAs are unessential. In rapidly dividing cells, including cancerous ones, the cell growth rate (Dilution factor) is the determinant factor for gene silencing duration; therefore, chemically modified siRNAs with longer intracellular half-life will not affect gene silencing activities [71].

### 3.2. Dosage Schedule

Unlike siG12D LODER, which is administered directly into the pancreas using a EUS needle, all investigational siRNA-mediated nanotherapeutics were parenterally administered via intravenous infusion. The dose of the incorporated siRNA monotherapies varies; DCR-MYC and Atu027 were administered with low doses of up to 0.3 mg/kg and 0.336 mg/kg, respectively, ALN-VSP02 and TKM-PLK1 doses were given up to 1.7 mg/kg, whereas siG12D LODER was administered at doses ranging from 0.025 to 3 mg. With the exception of CALAA-01 and siRNA-EphA2-DOPC, the doses of all investigational siRNA-based therapeutics were scaled according to body weight. siG12D LODER doses were scaled neither by body weight nor by body surface area. The frequent administration of anticancer siRNAs is obligatory to suppress more of the targeted genes, thereby ensuring successful outcomes, as evidenced by the Bartlett and Davis study [71]. The dosing schedule is carefully chosen, considering that gastronomic dosing administration is unnecessary for siRNA-based nanotherapeutics [72]. In addition, frequent administration of anticancer siRNAs nanotherapeutics is necessary to prevent chemotherapeutic resistance and metastasis; therefore, once to twice weekly dosing is desirable for siRNA nanotherapeutics with doses ranging up to 1.5 mg/kg [64]. CALAA-01, Atu027, siRNA-EphA-DOPC, and iExosomes were administered more frequently (twice-weekly dosing) as compared to once-weekly dosing involving TKM-PLK1, DCR-MYC and NBF-006. Although ALN-VSP02 was administered in the dose range of 0.1–1.5 mg/kg, it was only given once every two weeks. Tumor hypoperfusion induced by anti-VEGF siRNAs is expected to influence the cancerous cell doubling times and prolong the duration of gene knockdown. siG12D LODER was designed as slow and controlled release implants, releasing siRNA for up to 4 months. As a result, investigational siG12D LODER was administered as a single dose or repeated doses every four months. Besides, the antitumor effect of the siRNA nanotherapeutics may affect the frequency of administration. Some RNAi-mediated therapies induce knockdown targeted proteins resulting in cell growth arrest rather than cellular death; arrested cancerous cells will not be cycling, and the duration of gene silencing will be longer. It is anticipated that frequent administration of these therapeutics, considering safety limitations, may lead to cellular death [73]. Infusion time varied between different anticancer siRNAs nanotherapeutics. The infusion rate of Atu027 was slowest with over 4 h infusion, followed by DCR-MYC and EphA-siRNA-DOPC (infused for 2 h), infusion of CALAA-01 and TKM-PLK1 for 30 min, and infusion of ALN-VSP02 and iExosomes for 15 min. A rapid infusion rate is associated with infusion-related adverse events. Nevertheless, Atu027, which was infused over a long period, is the only investigational treatment administered with no pre-medication therapies (Table 3).

### 3.3. Pharmacokinetic Parameters

**Figure 4:**
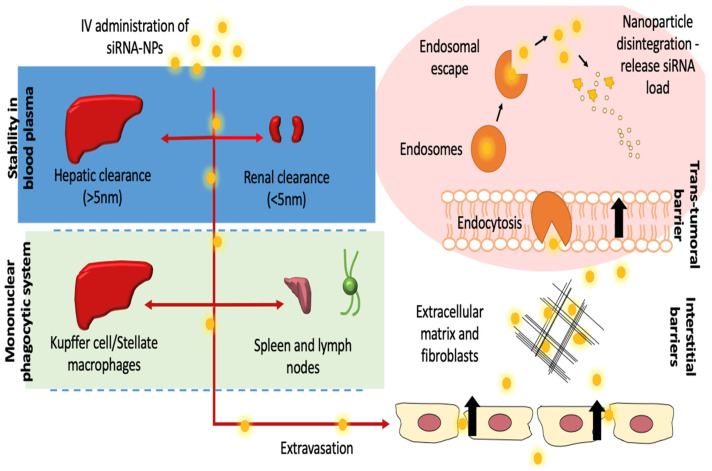
Physiological barriers to in vivo delivery of siRNA-nanoparticles. Extracellular barriers include hepatic and renal clearance, which are dependent on the particle sizes. Opsonization and macrophage engulfment via the mononuclear phagocytic system (MPS) further reduce the bioavailability of siRNA nanoparticles in the plasma. Extracellular matrix (ECM) trapping further decreases the localization of siRNA complexes into the tumor tissues. Intracellular barriers involve effective endosomal escape and siRNA release into the cytosol to initiate RNAi-associated gene knockdown activity.

Designing the size of nanoparticles is vastly important to determine the excretion pathway of the complexes. Particles of <5nm are primarily eliminated renally, whereas larger particles are excreted through the liver (Figure 4). Nanotherapeutics cleared either hepatically or by MPS are preferably dosed according to the body surface area [64]. LNPs are cleared mainly by the liver; specific biodistribution of these delivery systems into the liver and the spleen enhances systemic elimination. CALAA-01 is eliminated primarily by the kidney, and is not exposed to the MPS saturation.

Determining the maximum drug concentration (Cmax) and area under curve (AUC) is essential to determine the dose of nanotherapeutics in human trials. Dosing scale by body surface area is extensively used for cytotoxic agents; however, siRNA nanotherapeutic doses depend mainly on the clearance mechanism. Pharmacokinetics parameters (Cmax and AUC) for all investigational siRNA monotherapies were scaled by body weight. The Cmax and AUC were proportional to the administered dose with no accumulation following multiple administrations of investigated complexes. Dose accumulation is proposed to occur with therapeutics that saturate the MPS. Although CALAA-01 doses are scaled according to body surface area, Cmax and AUC are best extrapolated with dose scaling by body weight rather than body surface area.

### 3.4. Safety Profile

Phase I trials revealed that clinically investigated anticancer siRNAs were safe and well-tolerated in the tentative dose range. The maximum tolerated dose (MTD) was not determined for CALAA-01, ALN-VSP02, Atu027, DCR-MYC, and siG12D LODER. Patients treated with CALAA-01 experienced DLTs at different doses; therefore, no MTD was determined. DLTs were observed in two patients receiving 30 mg/m^2^ of CALAA-01 in phase 1a, and these patients were instantaneously withdrawn from the study. In the phase 1b trial, patients were recruited to receive 18 mg/m^2^ in cycle 1, followed by 27 mg/m^2^ in the subsequent cycles. Two out of five patients receiving 18 mg/m^2^ developed DLTs, and, as a result, the trial was terminated. Although DLT occurred in one patient receiving 0.336 mg/kg of Atu027, there is insufficient data to consider 0.336 mg/kg as MTD, and hence no MTD was documented in the interventional dose range of 0.001–0.336 mg/kg. No DLTs were documented in the dose range of 0.025–3 mg/kg after local implantation of siG12D LODER, and MTD was not determined. MTD was only determined for TKM-PLK1 treatment; the dose-escalation phases demonstrated no safety concerns concomitant with TKM-PLK1 administration at doses of less than 0.9 mg/kg, while 0.75 mg/kg was determined as MTD. A subsequent expansion phase trial reported grade 4 DLTs in two patients receiving 0.75 mg/kg; hence, MTD was amended to 0.6 mg/kg.

In many trials, the most common adverse events were confined to infusion-related adverse events and cytokine elevation-mediated symptoms. Serious infusion-related side effects that attracted investigators’ intervention were documented during CALAA-01 and ALN-VSP02 infusion: Grade 3/4 infusion-related side effects including hypersensitivity and fever accompanied CALAA-01 treatment, resulting in the infusion being halted. Preclinical studies confirmed hypersensitivity reactions during intravenous infusion of CALAA-01 in dogs and monkeys; therefore, antihistamines and steroids were included before CALAA-01 treatment, even though severe hypersensitivity reaction developed in only one patient [26]. Grade 2 infusion-related side effects reported in patients treated with ALN-VSP02 were managed by lengthening the infusion time to 30 to 60 min. Non-serious infusion-related side effects were observed following intravenous infusion of TKM-PLK1 and DCR-MYC therapies. Atu02 is the only systemic siRNA therapeutic infused with no pre-medication therapies. Intravenous infusion of Atu027 over an extended time was commonly accompanied by fatigue rather than infusion-related side effects. Various types of cytokines were elevated after CALAA-01, ALN-VSP02, and TKM-PLK1 treatment. Chills and rigors are cytokine release-mediated symptoms reported in patients treated with high doses of ALN-VSP02 and TKM-PLK1. No concomitant cytokine release symptoms were reported with CALAA-01. Cytokine level was not increased upon Atu027, DCR-MYC, and siG12D LODER administration. siRNAs induced complement component stimulation; CALAA-01, ALN-VSP02, and Atu027 administration resulted in mild and transient complement activation with no significant side effects. Complement component stimulation was not documented in TKM-PLK1, DCR-MYC, and siG12D LODER therapeutics. Gastrointestinal symptoms were the most common siG12D LODER-related side effects.

Hepatotoxicity and nephrotoxicity are other significant concerns related to nanoparticle delivery systems. Preclinical studies showed nephrotoxic effects associated with the administration of high doses of CALAA-01. On the contrary, no renal function deterioration was documented in the human clinical trial; this result was associated with lower doses being received and the employment of hydration protocols pre- and post-CALAA-01 administration. No abnormalities on the renal function tests in patients receiving 3 mg/kg siG12D LODER except for one patient who developed grade 2 renal failure, possibility attributed to the concomitant administration of chemotherapeutic agents (FOLFIRINOX). Dose-limiting hepatotoxicity was not reported with Atu027, TKM-PLK1, DCR-MYC and CALAA-1 treatments. Hepatotoxic effect in patients treated with LNP delivery systems was insignificant. No liver enzyme elevation was reported in all anticancer siRNAs nanotherapeutics’ trials. Although no clinically significant hepatic diseases accompanied ALN-VSP02 administration at dose range of 0.1–0.7 mg/kg, one patient with PNET and previous splenectomy and hepatectomy developed liver failure after receiving 0.7 mg/kg of ALN-VSP02, and subsequently died. A grade 3 transient AST elevation was reported in one patient receiving DCR-MYC treatment. Landen et al. revealed that EphA-siRNA-DOPC is substantially deposited in the liver, kidney, and lung with no significant cytotoxicity [56]. The result was comparable to Wagner et al., where multiple administration of EphA-siRNA-DOPC in non-human primates was not associated with significant hepatotoxicity [57].

Several siRNA nanotherapeutics manifested serious hematologic and electrolyte abnormalities. One patient developed severe anemia and two patients established lymphopenia following CALAA-01 administration. Two patients receiving 1.25 mg/kg ALN-VSP02 developed transient grade 3 thrombocytopenia that resolved without the need for platelet transfusion. A Grade 3/4 neutropenia and grade 4 pancytopenia were documented in siG12D LODER administration. Grade 3 hyponatremia and transient grade 3 hypokalemia were reported in patients infused with CALAA-01 and ALN-VSP02, respectively. Idiosyncratic and clinically significant adverse effects of Atu027 was frequently associated with elevated lipase level with no clinical pancreatitis. Serious adverse events including myocardial infarction, atrial fibrillation, and pulmonary edema were reported in three patients treated with TKM-PLK1, and severe GI side effects, primarily grade 3/4 cholangitis, grade 3 abdominal pain, and colonic obstruction, were noted in patients receiving siG12D LODER. iExososmes’ safety profile was established by Mendt and his colleagues, who confirmed that long-term administration of iExosomes was safe and not accompanied by off-target and/or immune-stimulating effects [52].

### 3.5. Antitumor Effect and Pharmacodynamics

**Figure 5:**
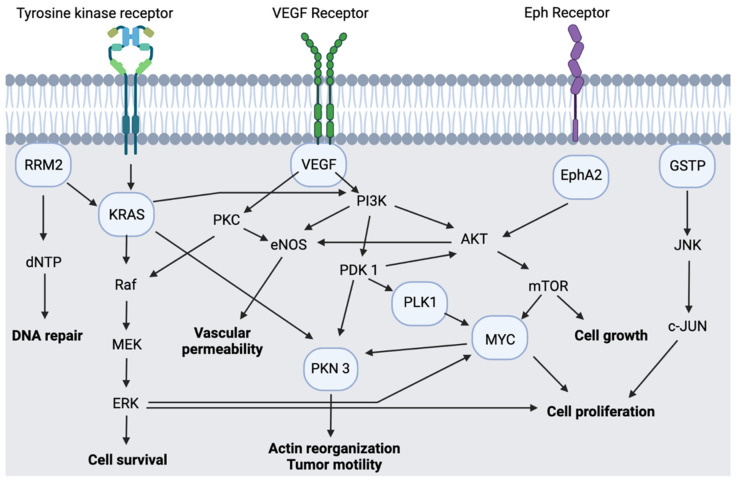
Simplified signaling cascade of selected genes for cancer therapy in clinical trials. Created with BioRender.com.

The phase I trials’ primary outcomes are to measure the safety profile and determine the maximum tolerated dose (MTD) and the DLT of the investigational therapeutics; thus, antitumor effect and pharmacodynamic data are limited. The gene silencing effect of all clinically investigated siRNA nanotherapeutics is achieved through RNAi mechanism of action and the formation of RISC, except for DCR-MYC, whose RNAi mechanism targets the Dicer, which causes knockdown of many gene pathways that promote cancer proliferation and survivability, as mentioned in Figure 5. In vitro studies demonstrated high potency of the anticancer agents; they induced suppression of the targeted genes/proteins at nanomolar (CALAA-01 and Atu027) to picomolar (ALN-VSP02 and DCR-MYC) concentrations, based on the IC_50_ values.

The primary clinical impact for most siRNA-loaded nanotherapeutics is tumor stabilization and partial response. Partial stabilization was attained in patients receiving ALN-VSP02, Au027, TKM-PLK1, and siG12D LODER treatment. The best clinical response achieved post-CALAA-01 administration is disease stabilization. Only one patient receiving ALV-VSP02 therapy achieved a complete response. Unlike Atu027 and siG12D LODER, all anticancer siRNAs were clinically examined as monotherapies, and favorable antitumor activity was demonstrated, excluding monotherapy of TKM-PLK1, where the antitumor efficacy was deemed unsatisfactory. Atu027 and siG12DLODER were investigated in the combination of gemcitabine alone or concomitantly with another chemotherapeutic agents. Simultaneous administration of Atu027 and siG12D LODER with SOC therapies exhibited a clinically promising antitumor effect.

### 3.6. Challenges of Ongoing and Future Human Trials

Human trials provided researchers with a plethora of information and paved the way to develop innovative delivery systems that will be immensely suited for clinical application. Animal studies could not substitute human trials, and their results are not easily extrapolated to humans. Therefore, clinical trials should be carefully optimized to attain more comprehensive and clinically acceptable results. Critical recommendations should be carefully deliberated upon in ongoing and future studies. Numerous limitations were and will continuously be encountered in the clinical trials; therefore, modification to ongoing studies should be encouraged. According to the trials discussed in this review, siRNA nanotherapeutics are relatively non-specific to tumor tissues with evident depositions in other organs. Patients involved in the clinical studies were not screened for gene mutations prior to the designated treatment. Even with the promising results, no proper correlation can be established between the antitumor activity and the patients’ clinical responses, as the data on antitumor activities and pharmacodynamics are limited.

### 3.7. Design Concerns for Future Development of RNAi-Mediated Anticancer Nanotherapeutics

The development of novel nanoparticle delivery systems and the optimization of siRNA formulations are vital not only to improve anticancer nanotherapeutics’ cellular uptake, but also to enhance their specificity and ensure therapeutic effectiveness. Numerous concerns upon developing new formulations for human trials should be weighed: (1) The production of larger quantities of nanoparticles (compared to small amounts in animals’ studies) may negatively impact nanoparticles’ behavior and function, aggregation, agglomeration, off-target issues and immunity; thus, safety limitations should be considered, and the production techniques of nanoparticles should be continuously improved. (2) Optimization and narrowing of nanoparticles’ diameter in the range of 20–30 nm; more homogenous size ranges showed better reproducibility and activity in humans [74,75,76]. (3) Simultaneous delivery of two or more siRNAs in the delivery systems may improve antitumor efficacy with no increase in toxicity and off-target effects. (4) Substitution of the standard nanoparticle vehicles with biodegradable nanoplatforms, as they exhibit a favorable safety profile. (5) Nanoparticles (and not siRNAs) could be functionalized using targeting ligands. While targeting may not enhance the uptake into tumor cells, targeting ligands may address other biological issues including intracellular trafficking and intracellular siRNA escape [77].

## 4. Conclusions

The discovery of the RNAi mechanism has paved the new way for targeted therapeutics with potential to treat many diseases including cancer. The remaining challenge that hampers the application of RNAi in vivo is the identification of the most suitable vehicle to ensure that siRNA is protected in circulation following intravenous administration, prior to internalization. Recent clinical trials on synthetic siRNA-mediated nanotherapeutics have demonstrated promising results towards the treatment of various carcinomas. Most clinical trials revealed that anticancer siRNA nanotherapeutics are safe, tolerable, and linked to mostly mild to moderate adverse effects. However, data on antitumor activities and pharmacodynamics are still lacking. Comprehensive investigation is vital to allow significant breakthroughs in siRNA nanotherapeutics and future commercialization of the therapy as part of the mainstream options in treating cancer patients.

## Figures and Tables

**Table 1:** Anticancer siRNA mediated nanoparticles in clinical trials.

Therapeutic Name	Indications	Target Gene/Protein	Route of Administration	Phase/Status	Reference
CALAA-01	Cancer, Solid Tumor	RRM 2	Systemic/IV infusion	Phase I/Terminated	[9]
ALN-VSP02	Solid Tumors	VEGF, KSP	Systemic/IV infusion	Phase I/Completed	[15]
Mesenchymal Stromal Cells-derived iExosomes	Pancreatic Cancer	KRAS G12D Mutation	Systemic/IV infusion	Phase I/Not yet recruited	[36]
siRNA-EphA2-DOPC	Advanced Cancers	EphA2	Systemi /IV infusion	Phase I/Not completed yet	[39,41]
Atu027	Advanced or Metastatic Pancreatic Cancer(II), Solid Tumors(I)	PKN3	Systemic/IV infusion	Phase II/Completed	[16,18,19]
TKM- PLK1 (TKM-080301)	Adrenal Cortical Carcinoma(II), Hepatocellular Carcinoma(II), Neuroendocrine Tumor(II), Solid Tumors(I)	PLK-1	Systemic/IV infusion	Phase II/Completed	[25,28,29]
siG12D LODER	Pancreatic Ductal Adenocarcinoma, Pancreatic Cancer	KRAS G12D mutation	Local/Surgical implantation	Phase II/Ongoing	[32]
DCR-MYC (DCRM1711)	Solid Tumors, Hepatocellular Carcinoma, Multiple Myeloma, NonHodgkins Lymphoma, Pancreatic Neuroendocrine Tumors	MYC	Systemic/IV infusion	Phase II/Terminated	[37]
NBF-006	Non-Small-Cell Lung, Colorectal, and Pancreatic Cancer	GSTP	Systemic/IV infusion	Phase I/Recruiting	[60]

Abbreviation: DOPC, 1,2-dioleoyl-*sn*-glycero-3-phospahtidylcholine; GSTP, glutathione-S-transferase P; i.v., intravenous; EphA2, EPH receptor A2 (ephrin type-A receptor 2); KSP, kinesin spindle protein; LNP, lipid nanoparticle; LODER, LOcal Drug EluteR; PKN3, protein kinase N3; PLK1, polo-like kinase 1; RRM2, ribonucleoside-diphosphate reductase subunit M2; siRNA: small interfering RNA; VEGF, vascular endothelial growth factor.

**Table 2:** Characteristics of siRNA motifs and delivery nanoplatforms within RNAi mediated anticancer nanotherapeutics.

Therapeutics’ Name	siRNA Characteristics			Delivery System				
	Encapsulation	Chemical Modification	Numbers of siRNA Motifs	Natural/Artificial	Type	Biodegra-Dability	Targeting	Targeting Ligand
CALAA-01	Encapsulated	Yes	Single	Artificial	Cyclo-dextrin based polymer	No	Yes	Human transferrin ligand
ALN-VSP02	Encapsulated	No	Two	Artificial	LNP	No	No	-
siRNA-EphA2-DOPC	Encapsulated	No	Single	Artificial	DOPC LNP	No	No	-
Mesenchymal Stromal Cells-derived iExosomes	Encapsulated	No	Single	Natural	Exosomes	No	Yes	CD47
Atu027	Encapsulated	Yes	Single	Artificial	AtuPlex Technology	No	No	-
TKM-PLK1 (TKM-080301)	Encapsulated	No	Single	Artificial	LNP	No	No	-
siG12D LODER	Encapsulated	No	Single	Artificial	LODER polymer	Yes	No	-
DCR-MYC (DCRM1711)	Encapsulated	No	Single	Artificial	EnCore LNP	No	No	-
NBF-006	Encapsulated	No	Single	Artificial	LNP	Yes	No	-

Abbreviation: DOPC, 1,2-dioleoyl-*sn*-glycero-3-phospahtidylcholine; EphA2, EPH receptor A2 (ephrin type-A receptor 2); LNP, lipid nanoparticle; LODER, LOcal Drug EluteR; RNAi, RNA interference; siRNA: small interfering RNA.

**Table 3:** Dosage schedule for the clinically investigated anticancer siRNA nanotherapeutics.

Therapeutics’ Name	Dosing Scale	Dose	Infusion Time	Dosage Frequency	Cycle	Pre-Medications	Maximum Tolerated Dose
CALAA-01	Body surface area	3–30 mg/m^2^	30 min	Twice weekly	21 days	Yes	Not determined
ALN-VSP02	Body weight	0.1–1.7 mg/kg	15 min	Once every two weeks	28 days	Yes	Not determined
siRNA-EphA2-DOPC	Body Surface area	0.45 mg/m^2^	2 h	Twice weekly	21 days	-	The trial not recruited yet
Mesenchymal Stromal Cells-derived iExosomes	Body weight	Not reported	15–20 min	Twice weekly	14 days	-	The trial is on-going
Atu027	Body weight	Phase II: 0.253 mg/kg	4 h	Twice weekly	28 days	No	Not determined
TKM- PLK1 (TKM-080301)	Body weight	Phase II: 0.6–0.75 mg/kg	30 min	Once weekly	28 days	Yes	0.6mg/kg
siG12D LODER	-	0.025–3 mg	-	Once monthly	-	No	Not determined
DCR-MYC (DCRM1711)	Body weight	0.1–0.3 mg/kg	2 h	Once weekly	21 days	-	Not determined
NBF-006	-	-	-	Once weekly × 4 weeks	Every 6 weeks	-	-

Abbreviation: DOPC, 1,2-dioleoyl-*sn*-glycero-3-phospahtidylcholine; EphA2, EPH receptor A2 (ephrin type-A receptor 2); LODER, LOcal Drug EluteR; siRNA: small interfering RNA.
